# Senescence and costs of reproduction in the life history of a small precocial species

**DOI:** 10.1002/ece3.5272

**Published:** 2019-05-29

**Authors:** Fritz Trillmich, Edda Geißler, Anja Guenther

**Affiliations:** ^1^ Animal Behaviour University of Bielefeld Bielefeld Germany; ^2^ Department of Evolutionary Genetics Max Planck Institute for Evolutionary Biology Plön Germany

**Keywords:** postnatal development and mortality, reproductive effort, reproductive seasonality, reproductive senescence, trade‐off

## Abstract

Species following a fast life history are expected to express fitness costs mainly as increased mortality, while slow‐lived species should suffer fertility costs. Because observational studies have limited power to disentangle intrinsic and extrinsic factors influencing senescence, we manipulated reproductive effort experimentally in the cavy (*Cavia aperea)* which produces extremely precocial young. We created two experimental groups: One was allowed continuous reproduction (CR) and the other intermittent reproduction (IR) by removing males at regular intervals. We predicted that the CR females should senesce (and die) earlier and produce either fewer and/or smaller, slower growing offspring per litter than those of the IR group. CR females had 16% more litters during three years than IR females. CR females increased mass and body condition more steeply and both remained higher until the experiment ended. Female survival showed no group difference. Reproductive senescence in litter size, litter mass, and reproductive effort (litter mass/maternal mass) began after about 600 days and was slightly stronger in CR than IR females. Litter size, litter mass, and offspring survival declined with maternal age and were influenced by seasonality. IR females decreased reproductive effort less during cold seasons and only at higher age than CR females. Nevertheless, offspring winter mortality was higher in IR females. Our results show small costs of reproduction despite high reproductive effort, suggesting that under ad libitum food conditions costs depend largely on internal regulation of allocation decisions.

## 1 INTRODUCTION

The assumption of trade‐offs between reproductive effort and self‐maintenance is central to life‐history theory (Roff, 2002; Stearns, 1992) and has been explained by the disposable soma theory (Kirkwood & Holliday, 1979). In this theory, allocation of essential resources to reproduction is predicted to reduce the options of females to maintain their own body condition, thereby increasing senescence and reducing future fecundity through physiological costs incurred (Fowler & Williams, 2017; Kirkwood & Rose, 1991; Speakman, 2008). This trade‐off will be harder to detect in long‐lived species, because they should be more restrained in allocating resources to a given reproductive episode. In long‐lived species, expectation of future reproduction, and thereby reproductive value at a given age, is higher than for short‐lived animals.

There are several possibilities how females can avoid such costs: (a) they may produce young of lower quality that have lower chances of growth and survival, thereby transferring costs to offspring. Such a strategy reduces maternal fitness, if not compensated by substantially better maternal survival or higher future fecundity prospects (Berghänel, Heistermann, Schülke, & Ostner, 2017; Love & Williams, 2008; Martin & Festa‐Bianchet, 2010). (b) Alternatively, females may reduce the costs of reproduction by producing smaller litters (potentially even with higher offspring quality). Which of these alternatives results in higher maternal fitness depends on the relative fitness of such offspring, that is, is a consequence of the quality–quantity trade‐off involved.

When investigating the relationship between reproductive effort and self‐maintenance under natural conditions (i.e., observational studies), several confounding factors may influence the observed trade‐off and mask potential patterns (Clutton‐Brock et al., 1996; Hamel et al., 2010). Differences in the general ability for resource acquisition and the consequent health status (often called “quality”) of females, for example, can affect the trade‐off. High‐quality females can afford to invest more into reproduction than low‐quality females and at the same time maintain their own good condition, masking the trade‐off between reproduction and self‐maintenance (van Noordwijk & de Jong, 1986). Such an effect has, for example, been shown by Weladji et al. (2006) for reindeer (*Rangifer tarandus*). Females may begin reproduction at different ages with potential consequences for future fertility and the process of senescence (Nussey, Kruuk, Donald, Fowlie, & Clutton‐Brock, 2006). Similarly, differences in resource availability can mask this trade‐off (de Jong & van Noordwijk, 1992). Differential access to resources may influence reproductive success and lead to a negative phenotypic correlation between reproductive success and the onset of senescence. Because of these potential interactions of several factors, trade‐offs can only be demonstrated convincingly by manipulating one of the involved traits (Hamel et al., 2010; Reznick, 1985).

Another aspect influencing this life‐history trade‐off is the reproductive strategy of a given species. Life histories differ greatly between species producing altricial and precocial offspring (Bielby et al., 2007; Ricklefs & Wikelski, 2002) perhaps most in a trait called “pace of life” by Ricklefs and Wikelski (2002). Among mammals, most species with precocial offspring are large, produce small litters or are even monotocous (Derrickson, 1992), have low mortality rates, and thereby follow a life history with a slow pace of life, whereas smaller species, exposed to high mortality, usually lead fast lives. Most of the latter species produce altricial young. For a large mammal producing few precocial offspring, the relative value of an offspring is likely higher than for a small mammal producing many altricial offspring. If so, mothers of precocial young may gain less by reducing the quality of offspring to protect their own reproductive value. Experimental studies of the onset of senescence and the cost of reproduction under controlled conditions are scarce (Hamel et al., 2010; Schubert et al., 2009) and are very hard to do with long‐lived mammals. In this respect, the cavy (*Cavia aperea*; average adult, full‐grown female mass about 680 g), the wild ancestor of the guinea pig, is a particularly interesting rodent since it possesses a puzzling combination of life‐history traits: Cavies are short‐lived (Kraus, Thomson, Künkele, & Trillmich, 2005), but produce small litters of large, extremely precocial young after a very long pregnancy for their size—60 days (Kasparian, Geißler, & Trillmich, 2005; Kraus, Trillmich, & Künkele, 2005; Künkele, Kraus, & Trillmich, 2005). The species shows high reproductive effort and near‐continuous reproduction in the wild. Reproduction has been studied in the field (Asher et al., 2008) as well as in captivity. Potentially, mature females reproduce continuously, since they have a postpartum estrus and may therefore be always pregnant once they have started reproduction (Rehling & Trillmich, 2008a). Seasonality strongly influences age at maturity (Guenther, Palme, Dersen, Kaiser, & Trillmich, 2014; Trillmich, Mueller, Kaiser, & Krause, 2009) and sexual physiology and behavior (Hribal, Rübensam, Bernhardt, Jewgenow, & Guenther, 2018; Rübensam, Hribal, Jewgenow, & Guenther, 2015).

To determine the consequences of differences in reproductive effort, we established two groups of cavy females in outdoor enclosures. One group always had males available and, therefore, could potentially reproduce continuously (CR), and the other group had access to males only intermittently and thus had periods of rest between reproductive episodes (intermittent reproduction; IR). To assess long‐term consequences of this experimentally induced difference in reproductive activity, the experiment ran for 3 years, longer than the expected lifetime of a free‐living cavy (average survival under natural conditions 3–16 months; Kraus, Thomson, et al., 2005). This setup also allowed to study the influence of seasonality in photoperiod and low temperature during the cold season (which is the most difficult season for free‐living animals) on female reproductive effort and early pup development.

Assuming that reproduction is costly (Roff, 2002; Stearns, 1992), we predicted that the CR group would show a higher rate of female senescence and produce smaller litters or litters of smaller young than the IR group. We know that female guinea pigs and wild cavies react very little to increases in offspring demand (Laurien‐Kehnen & Trillmich, 2003; Rehling & Trillmich, 2008b). Therefore, we also tested whether aging females transferred some of the costs of reproduction to the offspring. If so, we expect offspring mortality to increase with female age and to be higher and growth to be slower in the CR than the IR group as a consequence of the former's potentially continuous reproductive output. Moreover, we predicted an influence of seasonality on female reproductive effort, with reduced reproduction during the cold season (Rübensam et al., 2015) and more so in the CR group.

## 2 MATERIALS AND METHODS

Wild cavies (*C. aperea*) are a species of South American rodent distributed from southern Brazil to northern Argentina. In the wild, they reproduce throughout the year with less reproductive activity during the winter months, when temperatures regularly fall to around 0°C. After a pregnancy of 60 days, females produce small litters of 1–10 extremely precocial young, with a mean of about 2.3 (Kasparian et al., 2005; Rood & Weir, 1970). Young are weaned when about 19 days old (Rehling & Trillmich, 2008a). Females mature briefly after weaning when 30–70 days old (Trillmich, Laurien‐Kehnen, Adrian, & Linke, 2006) and potentially reproduce from then on uninterruptedly as they mate during a postpartum estrus. Mean survival in the field of the closely related dark‐backed cavy (*Cavia magna*) was estimated to be 3–16 months (Kraus, Thomson, et al., 2005). Predation plays the major role in mortality (Kraus & Rödel, 2004). Experimental animals were derived from wild animals captured in northern Argentina and southern Uruguay. They were randomly bred (avoiding sibling matings) at Bielefeld University since the 1980s. Fresh wild animals were imported repeatedly from northern Argentina and Uruguay to reduce inbreeding effects.

### 2.1 Climatic conditions

To provide a natural, seasonal temperature regime, cavies were kept in outdoor enclosures, exposed to the local shifting climatic conditions (Figure S1). A shelter attached to the enclosure was kept at around 5°C by a dark heat lamp. The animals were often observed outside even at lower temperatures. In their natural environment in Uruguay, cavies experience comparable temperature shifts between summer and winter and are quite regularly exposed to winter temperatures below freezing. To characterize ambient conditions, temperature was measured in the open in the enclosures by means of I‐buttons (Dallas Semiconductors) that recorded temperature every 10–30 min. The seasons are categorized following the meteorological system as spring (March–May), summer (June–August), autumn (September–November), and winter (December–February). During 2006, minimal temperature was −8°C in January and maximum was 37°C in July; the yearly mean was 11.1°C.

### 2.2 Experimental setup

Groups were established between May 2003 and May 2004, and the experiment ran for 36 months. No groups were established in the period from October 2003 to April 2004 to avoid the start of a group during the cold time of the year. Thirty‐day‐old females were placed into a given enclosure within 1–14 days (mean 7 days) until a group of three animals of similar size had become established. Outside enclosures measured 230 × 480 cm and were connected to a shelter of 100 × 170 cm surface area. Animals were fed pellets (Höveler Guinea pig chow) and hay ad libitum and received carrots three times a week and during summer occasionally fresh willow twigs. Water was always available and was supplemented once a week with 1g/L of vitamin C.

### 2.3 Continuous reproduction (CR)

Three 30‐day‐old, unrelated females were placed with a full‐grown, unrelated (*r* < 1/4) adult male in each CR outdoor enclosure. They stayed in the company of an adult male, who was exchanged every 30 days, for three years. Six groups of altogether 18 females were established in this way. One female died before producing a first litter, was not replaced, and ignored in the analyses. The experiment ended—for a given female—after three years or at death, whichever occurred first. Six of the 17 reproducing females died before the end of the experiment, and the earliest death occurred after 420 days (Table S1).

### 2.4 Intermittent reproduction (IR)

Again, three 30‐day‐old, unrelated females were placed with a full‐grown, unrelated (*r* < 1/4) adult male. They were kept in male company initially for 91 days, but by mistake in two enclosures for 139 and 146 days. Thereafter, females were left without a male for 63 days after which a male was placed in their enclosure for 91 days. As stated above, pregnancy of cavies lasts 60 days. We kept these animals with males for 91 days to allow for impregnation after a first litter had been produced. The 60 days without males was chosen to permit a period without males equal in length to a pregnancy. This alternation was repeated for the whole period until the end of the third year. As for the CR condition, six groups of altogether 18 females were established in this way. The experiment ran for 3 years and ended as for the CR group. Six of the 18 females died before the end of the experiment, and the earliest death occurred after 426 days (see Table S1).

In both, the CR and IR groups, males were exchanged once a month by moving them to the next enclosure. This ensured that males were also exchanged between CR and IR groups in order to average out any possible influence of individual males. Overall 12 males were exchanged between enclosures and replaced when they were older than 3 years or appeared sick.

CR and IR groups were always established simultaneously and treatment alternated between adjacent enclosures. In both experimental conditions, females that died were not replaced. Enclosures were checked daily to record births and deaths. When a litter was found, the mother's mass was determined and all pups (whether dead or alive) were sexed and weighed. Analyses included maternal mass only, if a given female was not pregnant at the time of measurement, otherwise we used postpartum mass so that effects of pregnancy on maternal mass were always excluded.

In rare instances, we could not determine sex and birth mass of a pup, because it was found partially eaten. We included the dead pup(s) in the recorded litter size. Pups were removed from the enclosures when 30 days old, that is, clearly after weaning. Every first weekday of a given month, all females were weighed and their skull length (from tip of nose to back of skull), which grows continuously during the first year, measured with calipers to the nearest tenth of a millimeter to determine maternal condition (skull length in mm/mass in g). If a female was not pregnant at the time of measurement, we used these values directly, otherwise skull length and postparturition mass were linearly interpolated between the two closest measurement time points.

Due to simultaneous parturitions, mothers of young of 14 litters (6% of all litters) of the CR group and of 26 litters (13% of all litters) of the IR group could not unequivocally be determined. In these cases, an equal number of pups were ascribed to both mothers, if both had lost about the same mass and the pup number was even, or the female with the higher mass loss was ascribed one or two more young, depending on mass loss of the females involved. Premature births (early abortions) and embryos found in postmortem examinations were not counted among the litters of a given female.

Reproductive effort was defined as litter mass at birth in relation to maternal mass at parturition (litter mass/maternal mass).

### 2.5 Statistical procedures

Data were analyzed using the free software R (version 3.3.1; R Developmental Core Team, 2016). We used a proportion test to analyze, if survival to the end of the experiment differed between treatment groups. A survival analysis with censoring was used to test for treatment differences in maternal age reached throughout the three‐year experimental period. A *t* test was employed to test for differences in litter size between litters conceived in a postpartum estrus and after a pause in reproduction. Modeling maternal age in a mixed model including “enclosure” as random effect produced the same results (results not shown).

To analyze reproductive parameters of females, we included treatment (two levels), season (four levels), maternal age, and maternal age^2^ as fixed effects in our linear mixed‐effect models (package lme4). In addition, we fitted the two‐way interactions of treatment with maternal age as well as treatment and season. Maternal ID was included as random effect to account for repeated measurements of the same female, but also to take into account potential differences in maternal quality. To analyze growth to maximum mass, we calculated a mixed‐effect model in which the relative difference (% of maximum body mass) to maximum mass reached throughout the experiment was fitted as response variable following Douhard, Gaillard, Pellerin, Jacob, and Lemaître (2017). We used a piecewise function in which a mass difference was only considered when the difference to maximum mass was positive, that is, when the animal still grew while mass loss was assumed to be absent before reaching maximum mass. Maternal age, treatment, and their interaction were fitted as fixed effects, and female ID was fitted as a random effect. The onset and rate of senescence, that is, loss of body mass, were modeled in the same way. Here, the relative loss of body mass compared to maximum body mass was used as response variable.

Models analyzing offspring data (litter mass, birth mass, early growth) included the same fixed‐effects structure. Paternal ID was fitted as a second, crossed‐random effect in addition to maternal ID. Residuals of the models were visually inspected for homogeneity of variances and normal distribution by using Q‐Q plots. Statistical significance of interactions was assessed by conducting likelihood‐ratio tests between full and reduced models (Zuur, Ieno, Walker, Saveliev, & Smith, 2009).

Perinatal mortality was analyzed using a generalized linear mixed‐effect model with binomial error structure. The structure of fixed and random effects was the same as previously described. To analyze postnatal mortality, stillborn offspring were excluded from the data. Hence, postnatal mortality is statistically independent of perinatal mortality. We used the same model structure to analyze pre‐ and postnatal mortality.

We present means and standard errors of raw data throughout the manuscript, if not stated otherwise.

## 3 RESULTS

### 3.1 General features of reproduction

**Figure 1:**
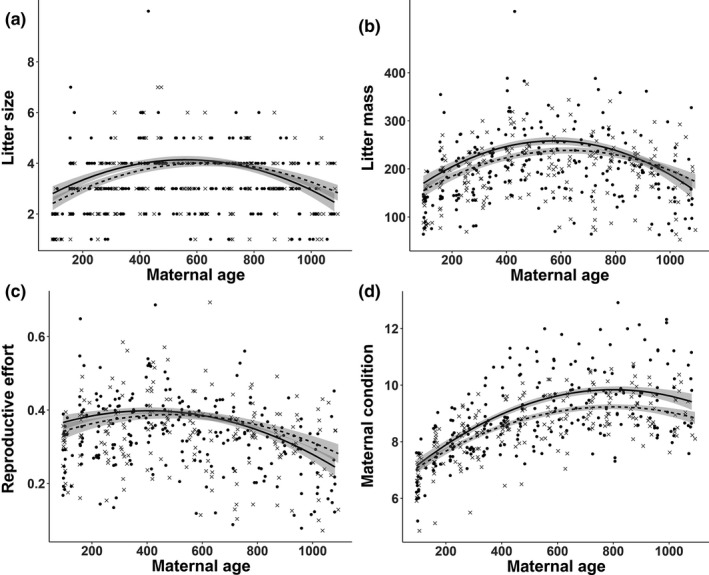
Effects of maternal age on continuously (CR) versus intermittently (IR) reproducing females. Black dots and solid lines refer to CR females and crosses and dotted lines to IR females. Gray bands around the lines represent 95% confidence intervals of regression lines. (a) Litter size with age; (b) litter mass (sum of mass of all pups) with age; (c) reproductive effort (litter mass/maternal mass) with age (d): maternal condition (skull length in mm/mass in g) with age

In both experimental groups, females conceived successfully when around 40 days old, at about 37% of their maximal postpartum mass (mean ± *SD*: 253 ± 33g CR; 248 ± 45g IR; whenever we refer to mass in the following this refers to nonpregnant or immediate postpartum mass). They grew substantially in mass and skull length during this first pregnancy and had reached about 63% of their maximum body mass (Table 2), when they first gave birth at about 100 days of age. First litters were smaller and lighter than prime‐age litters (Figure 1a,b), but represented roughly the same reproductive effort (RE; about one third of maternal mass; Figure 1c; Table 1) as in prime‐age females. While reproducing, female condition increased and, similar to female mass, leveled off at the age of 1.5–2 years (Figure 1d). The most productive female gave birth to 66 pups in 16 litters over the three years, corresponding to 4.4 times her own maximal body mass. Pups weighed around 60–65 g at birth and grew at a rate of about 5 g/day. Season strongly influenced these parameters (Table 1), and all the aforementioned parameters declined in females older than 600–800 days (Figure 1a–d).

**Table 1:** Comparison of seasonal changes in reproductive characteristics of wild cavies between the two experimental groups. Pup perinatal mortality refers to the mortality within a day of birth, postnatal mortality to the subsequent mortality until day 30

Reproductive parameter		Spring (March–May)	Summer (June–August)	Autumn (September–November)	Winter (December–February)	P season	P season * treatment
Maternal condition[mass (g)/skull length (mm)]	CR	8.95 ± 0.15	8.77 ± 0.26	8.80 ± 0.19	8.78 ± 0.18	<0.001	0.003
	IR	8.72 ± 0.14	8.59 ± 0.15	8.42 ± 0.17	8.43 ± 0.13		
Reproductive effort [litter mass/maternal mass]	CR	0.34 ± 0.02	0.35 ± 0.01	0.34 ± 0.01	0.30 ± 0.01	0.01	0.01
	IR	0.34 ± 0.02	0.34 ± 0.02	0.33 ± 0.01	0.32 ± 0.02		
Litter size	CR	3.3 ± 0.18	3.5 ± 0.19	2.9 ± 0.18	2.8 ± 0.14	<0.001	<0.001
	IR	3.4 ± 0.19	3.3 ± 0.20	3.2 ± 0.18	2.6 ± 0.14		
Litter mass [g]	CR	204.2 ± 10.0	215.4 ± 10.8	185.2 ± 10.0	184.4 ± 9.4	0.001	0.003
	IR	209.8 ± 10.2	198.5 ± 9.4	201.2 ± 9.9	180.9 ± 8.0		
Pup birth mass [g]	CR	63.5 ± 0.81	61.9 ± 0.89	63.64 ± 0.79	68.11 ± 0.84	<0.001	0.19
	IR	60.6 ± 1.0	61.9 ± 1.05	62.09 ± 1.27	64.42 ± 1.34		
Pup perinatal mortality [%]	CR	9.2	8.9	10.5	4.3	0.004	0.007
	IR	11.7	11.3	12.5	16.08		
Pup postnatal mortality [%]	CR	15.3	13.1	10.0	9.6	<0.001	<0.001
	IR	7.1	9.1	3.8	17.5		
Pup growth [g/day]	CR	5.18 ± 0.19	5.32 ± 0.20	4.95 ± 0.18	4.84 ± 0.17	<0.001	0.022
	IR	4.81 ± 0.21	5.18 ± 0.19	4.82 ± 0.17	4.91 ± 0.11		

Note

Presented are means ± *SEM* except for pup mortality, which is indicated in percent. P* season* indicates the significance of the main effect of season in the statistical model, while P season*treatment indicates significance of the interaction effect. CR = continuous reproduction; IR = intermittent reproduction.

### 3.2 Effects of continuous (CR) versus interrupted (IR) reproduction

#### 3.2.1 Effects on development of females

**Figure 2:**
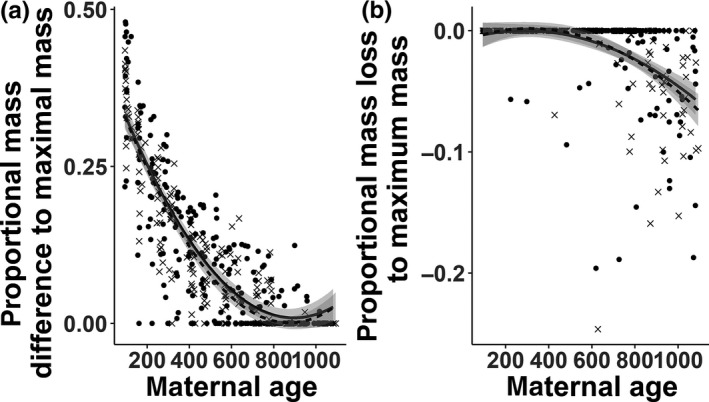
(a) Relative growth (proportional difference to maximum mass reached throughout the experiment) of CR (black line) and IR (dashed line) females. (b) Relative rate of senescence measured as mass loss compared to maximum mass reached throughout the experiment

During their lifetime, females of the CR group reached an average maximum mass of 688 g, those of the IR group of 655 g (Table 2). While this maximal nonpregnant mass did not differ significantly between CR and IR females (*t* = −1.02, *p* = 0.32), CR females were on average slightly heavier than IR females across their whole lifetime (see Figure S2, *t* = 4.61, *p* < 0.001). Females of the CR and IR group grew to their maximum mass at similar rates and reached it at a similar age (interaction experimental treatment × female age: *t* = 0.93, *p* = 0.36; Figure 2a).

**Table 2:** Means (±*SEM*) of life‐history parameters for the two groups of females. Tested by *t* tests (two‐tailed) unless stated otherwise

	Continuous reproduction	Intermittent reproduction	Group difference *p*
Age at 1st parturition	104 ± 2.4	103 ± 1.9	0.76
Mass at 1st parturition	422 ± 12.7	423 ± 13.0	0.93
Max. maternal mass (g)	688 ± 26.3	655 ± 18.5	0.31
Mean interlitter interval (days)	66.2 ± 1.9	86.2 ± 4.2	0.0002
Total litters/female	13.0 ± 0.98	10.9 ± 0.65	0.09
Mean litter size	3.14 ± 0.09	3.09 ± 0.09	0.045
Onset of senescence in body mass (days)	636 ± 56	707 ± 41	0.86
Onset of senescence in litter size (days)^a^	557	625	
Onset of senescence in litter mass (days)^a^	569	626	

a Estimates were calculated from statistical model instead of raw data due to strong fluctuations of litter size and mass across seasons.

Similar to absolute mass, maternal condition increased with female age for nearly two years before reaching a plateau (Figure 1d; *t* = 32.9, *p* < 0.001). While female condition did not differ at the start of the experiment, CR females increased condition more steeply with age as indicated by a significant interaction between age and treatment and maintained a better condition until the end of the experiment after three years (Figure 1d; *t* = 7.04, *p* < 0.001; for mass see Figure S2).

#### 3.2.2 Effects on female reproduction

**Figure 3:**
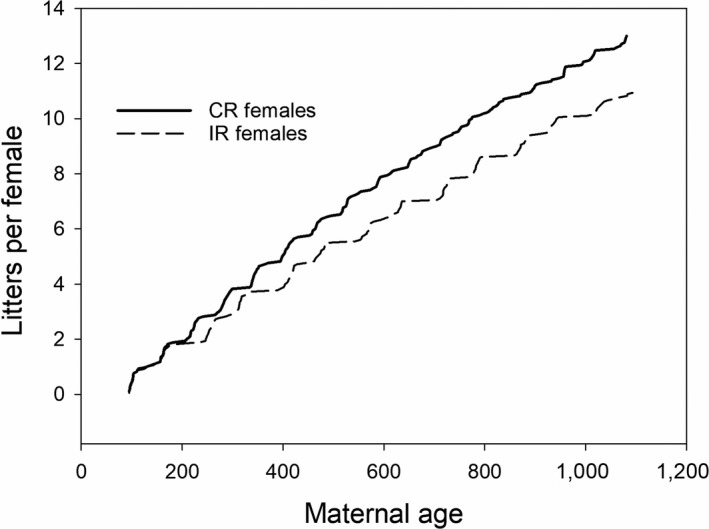
Cumulative number of litters per female. Over the duration of the experiment, CR females produced on average 13 litters per female, IR females 10.9

Overall females produced about 33% of their body mass in a given litter. Females in the CR and IR groups gave birth to their first litter at about the same age and body mass (Table 2, at 61.2% and 64.6% of their maximal mass, respectively). Mean interlitter interval for the 17 females of the CR group was significantly shorter (Table 2; min 60.25, max 94.2 days) than for the 18 females of the IR group (varying between 76 and 154 days), demonstrating that intermittent male removal led to more extended pauses between pregnancies for females of the IR group. The 17 females of the CR group produced 221 litters (694 pups; 351 males; for three pups, sex could not be determined) and the 18 of the IR group 197 litters (i.e., 15.8% fewer litters per female; 609 pups; 309 males; for 12 pups, sex not determined) over the three years of the experiment (Table 2; Figure 3). Overall mean litter size (calculated as mean of all litters in the respective group) was significant, if only marginally higher in the CR group (*t* = 2.03, *p* = 0.045; mean litter size 3.14 ± 0.09 in the CR group and 3.09 ± 0.09 in the IR group; without inclusion of simultaneous litters: 3.14 in the CR‐ and 3.04 in the IR group). In both groups, litters conceived in postpartum estrus were larger than litters conceived after a reproductive pause (postpartum 3.43 ± 1.25 *SD* pups, *n* = 175; vs. 3.09 ± 1.20 *SD*, *n* = 205 pups in a nonpostpartum conception; *t* = 2.753, *p* = 0.006, excluding first litters; no difference between CR‐ and IR group). Excluding first litters again, in the CR group, 169 of 204 (82.8%) litters were conceived postpartum, in the IR group 78 of 178 (43.8%). Postpartum conceptions in the IR group happened, when a female gave birth during the 63 days that a male was present.

#### 3.2.3 Effects of age/senescence

When analyzing potential differences between the two groups with respect to effects of aging, we found that the onset (table 2, *t* = 0.17; *p* = 0.86) and rate of senescence in body mass did not differ between CR and IR females (*t* = −0.25, *p* = 0.80; Figure 2b).

We observed a significant interaction of maternal age and experimental group in reproductive effort: RE did not differ between treatment groups up to a maternal age of about 600 days, thereafter CR females showed a stronger decrease in RE than IR females (Figure 1c, *t* = −3.6, *p* < 0.001). In both experimental groups, litter size initially increased with female age (and mass) and then declined again (Figure 1a). Litter size increased more steeply in CR females (*t* = 2.41, *p* = 0.015) but started to decline again as a sign of reproductive senescence earlier compared to IR females (Table 2). Litter size of CR females also decreased a little more steeply with age (Figure 1a; *t* = −8.1; *p* < 0.001). Litter mass was highly correlated with litter size (R = 0.89) and decreased more steeply in CR females compared to IR females (Figure 1b; interaction term experimental condition × maternal age, *t* = 2.49, *p* = 0.01). These data indicate a more pronounced reproductive senescence in CR females.

Six females in each group died during the course of the experiment (mean: CR 635 days; IR: 750 days; *t* = 0.414, *p* > 0.05; Table S1). Thus, survival was not demonstrably affected by experimental condition (*df* = 1; *Χ* ^2^ = 0; *p* = 1).

#### 3.2.4 Effects of seasonality

Female body mass and condition were affected by season (Table 1; *df* = 3; *Χ* ^2^ = 33.2; *p* < 0.001). As shown in Table 1, during autumn and winter, IR females decreased their reproductive effort less strongly than CR females. Litter size differed between seasons (Table 1) as found in earlier studies (Rübensam et al., 2015). Females had smaller litters in the cold seasons autumn and winter (*t* = −4.9, *p* < 0.001) and larger litters in summer (*t* = 2.6; *p* = 0.01).

#### 3.2.5 Effects on offspring

Birth mass of pups was unaffected by experimental group (*t* = 0.51, *p* = 0.61), maternal age (*t* = −0.11, *p* = 0.92), or their interaction (*t* = −1.36, *p* = 0.17). Birth mass differed across seasons as found in earlier studies (Rübensam et al., 2015; Table 1), but there was no interaction of experimental group and season.

Pup growth rate (mean: 5.0 g/day in the first 30 days of life) did not differ between experimental groups (*t* = 1.19, *p* = 0.24) and was not affected by maternal age (*t* = −0.26, *p* = 0.79) or the interaction of maternal age and experimental treatment (*t* = −0.59, *p* = 0.55). Growth rate differed across seasons (Table 1; *df* = 3; *Χ* ^2^ = 35.2; *p* < 0.001). In addition, there was a significant interaction between season and experimental treatment (*df* = 3; *Χ* ^2^ = 7.9; *p* = 0.04, Table 1), indicating higher offspring mortality in winter in the IR group.

Perinatal mortality was generally low (10.6%; Table 1) but increased with maternal age (*z* = 3.71, *p* = 0.002, binomial model). The interaction between maternal age and experimental group was not significant (*z* = −0.17; *p* = 0.87; Figure S3). Perinatal mortality was not predicted by pup birth mass, but very small and very large offspring tended to suffer higher mortality at birth.

Another 10.7% of pups born alive died before reaching 30 days of age. Postnatal mortality until day 30 was generally lower in the IR group (Table 1; *z* = 520, *p* < 0.001; Figure S3) but increased with increasing maternal age after 600 days of age for both groups (*z* = −4.9, *p* < 0.01). Offspring with a low birth mass suffered higher postnatal mortality (*z* = −4.25, *p* < 0.001).

Pup mortality was strongly influenced by season (Table 1, *z* = 2.44, *p* = 0.014) and showed an interaction between season and experimental treatment (*X* ^2^ = 9.4, *p* = 0.02). While postnatal mortality was lower in offspring of IR females in spring (*z* = −326.1, *p* < 0.001), summer (*z* = −83.3, *p* < 0.001), and autumn (*z* = −520.3, *p* < 0.001), it was significantly higher in winter (*z* = 270, *p* < 0.001) than for offspring of CR females. We have no explanation for this reversal of relative mortality under stressful temperature conditions.

## 4 DISCUSSION

Our experimental manipulation demonstrated that increased reproduction results in earlier senescence in some reproductive traits of cavies. However, we could not show the expected decrease in survival in the CR group. Contrary to our expectation, females in the CR group grew faster and maintained a higher body mass across most of their lifetime than females in the IR group. Reproductive effort, litter size, and litter mass decreased more steeply in old females of the CR than in the IR group. Birth mass and growth rate of pups showed no differences between experimental groups and did not change with maternal age. However, perinatal mortality of pups increased with maternal age, but again did not show a difference between CR and IR females. Overall, we found support for the predicted trade‐off between early and late reproduction that was also found in earlier studies (Lemaître et al., 2015). Despite finding several significant differences between continuous and interrupted reproduction, however, most effect sizes were small and indicated only slight differences between groups.

Our study excluded the influence of variability in resource abundance and acquisition that may obscure the detectability of costs of reproduction (van Noordwijk & de Jong, 1986). Ad libitum food conditions are expected to reduce mortality (Tidière et al., 2016). Reduction in the variability of food acquisition is also expected to decrease the effects of reproductive effort on senescence (Lemaître & Gaillard, 2017; Tidière et al., 2016). Nevertheless, the difference between the CR and IR groups in terms of body mass and reproductive senescence demonstrates the expected effect of a higher RE on aging. We are also aware that with this approach we can only study the phenotypic response of the species to differences in reproductive effort and not the underlying genetic trade‐off between life‐history traits (Reznick, 1985). However, our approach evaluates an important aspect of phenotypic trade‐offs.

Our manipulation of access to males for mating proved effective in reducing female reproductive output, but less so than we expected. This is partly explained by the unexpected finding that many females in the IR group entered estrus shortly after they had access to a male again, that is, estrus appeared to be induced by the presence of a male. This effect has not been described in cavies previously. In addition, females that gave birth during the presence of a male could conceive again during postpartum estrus and produce a litter during the subsequent period of male absence. Together, these effects explain the low difference between our experimental groups.

### 4.1 Effects on females

The lack of differences in survival between the CR and IR groups cannot be interpreted as evidence of a lack of survival costs of reproduction because our sample size was too small to detect medium or small effects on differential survival. In addition, as explained above, differences in reproduction between the two groups were smaller than originally envisaged which may have further reduced the detectability of potential differences. This agrees with the analysis by Ricklefs and Cadena (2007) who found no indication of a trade‐off between reproductive effort and life span in captive environments.

Contrary to our prediction, CR females reached a higher body condition and maintained higher mass than females of the IR group. Intermittent reproductive rests apparently did not increase female growth and maximal body mass. This may indicate a positive effect of the hormonal and morphological (organ remodeling; Speakman, 2008) changes accompanying pregnancy and lactation on body mass development as females of the CR group spent more of their lifetime in the pregnant and lactating state.

We detected reductions in reproductive effort in our study species only at an age when most free‐living females of the closely related species *C. magna* will have died already (Kraus, Thomson, et al., 2005). We assume that *C. aperea* suffers similar mortality as it lives in near‐identical habitat and is exposed to the same predators. Generation time was shown to be the best predictor of the onset of senescence in mammals and birds (Jones et al., 2008). Given that cavies in the field are expected to have a generation time less than 16 months (Kraus, Thomson, et al., 2005), the late onset of senescence in our experiment may be an effect of ad libitum food conditions in captivity. If aging rate in the field were higher, reproductive senescence as documented here may well occur during the expected lifetime of a cavy. The decrease in litter size (and the closely correlated litter mass) of old cavies was not caused by a selective effect, that is, that females with lower litter sizes and therefore lower reproductive effort survived longer, because litter sizes did not differ between females that survived until the end of the experiment and those dying earlier.

The stronger decrease in litter size and—correlated with it—litter mass of CR than IR females toward the end of the experiment indicates that senescence affected CR females slightly more than IR females. This effect also showed in the stronger reduction in RE in the CR group.

Similar decreases in litter size of old females have been observed in multiple species (alpine marmots, *Marmota marmota*, Berger, Lemaître, Gaillard, & Cohas, 2015; North American red squirrel, *Tamiasciurus hudsonicus*, Descamps, Boutin, Berteaux, & Gaillard, 2008; *Peromyscus leucopus*, Havelka & Millar, 2004; meerkat, *Suricata suricatta*; Sharp & Clutton‐Brock, 2010), but not in Columbian ground squirrels, *Spermophilus columbianus,* Broussard, Risch, Dobson, & Murie, 2003). As in the study of Berger et al. (2015), birth mass of individual pups did not decrease with maternal age.

### 4.2 Effects on offspring

Offspring perinatal and early postnatal mortality increased past maternal age of about 600 days despite a decline in litter size indicating senescence of old females in both groups. Increased offspring mortality decreases the energetic load on the female which is highest during the short lactation period (Speakman, 2008). At the same time, it reduces maternal fitness but decreases the fitness of the offspring to zero. A decrease of physiological efficiency caused by senescence is likely involved, so that a given energy allocation to reproduction is more costly for old females than for prime‐aged ones. Females did not jeopardize survival or future reproduction, but instead diminished their reproductive effort and the quality of some of their offspring, as observed in large herbivores with a slow life history (Clutton‐Brock et al., 1996; Hamel et al., 2010; Martin & Festa‐Bianchet, 2010). In contrast, growth of the remaining young was not affected by female age. Whether this is an effect of the smaller litter size of older females, of greater experience in pup rearing, or only a consequence of the early death of unfit offspring cannot be decided. The increased pup mortality may indicate that even in these short‐lived rodents, costs of reproduction were transferred to offspring as expected for large mammals (Clutton‐Brock et al., 1996; Gaillard & Yoccoz, 2003).

### 4.3 Life‐history trade‐off or physiological regulation?

The limited effects of our manipulation on the trade‐off between early and late reproduction and overall reproductive effort may reflect the constant availability of ad libitum food under captive conditions. Another explanation could be that reduced reproductive output due to unavailability of mates would be compensated for by increasing and/ or extending care for the offspring available, thereby decreasing the reproductive difference between treatment groups. For example, Verhulst, Tinbergen, and Daan (1997) demonstrated in great tits that removal of second clutches led to an extension of parental care for the first brood. A similar effect in our study is unlikely to have happened as parental care in cavies is rather limited and short lasting due to the extremely precocial nature of the young (Laurien‐Kehnen & Trillmich, 2003; Rehling & Trillmich, 2008b). In addition, such a difference should have been detectable in growth or postnatal mortality rates of young. However, we found no differences in these traits between CR and IR offspring.

The rather weak evidence for the trade‐off between early and late reproduction contrasts strongly with the clear seasonality effects which we observed despite continuous ad libitum feeding and the opportunity to avoid the harshest consequence of seasonal temperature changes by use of the mildly warmed retreats we offered. Even though the animals lived under ad libitum conditions, the low winter temperatures put some (energetic?) stress on females and showed up in decreased litter size, pup growth, and increased offspring mortality in winter, particularly in the IR group that reduced reproductive effort slightly less than the CR group. It seems that the winter effects depended more on photoperiod influences on physiological and reproductive systems than energetic limitations. Similar reductions in litter size have been observed before together with other physiological changes in cavies kept outdoors (Hribal et al., 2018; Rübensam et al., 2015). Given that we were able to observe this seasonal regulation so clearly, but documented comparatively mild costs of reproduction during the expected lifetime of the animals and only limited senescence effects late in life despite continuous reproduction in the CR group argues against major costs of reproductive effort in cavies.

This suggests that much of the short‐ and long‐term effects of reproduction on female reproductive effort and senescence we observed can be understood in terms of regulatory changes as suggested in the Y‐model of Harshman and Zera (2007). In this model, allocation is influenced by nutrient input sensing and signaling that influences somatic state and function as well as the level of reproduction. For example, in both groups, females that conceived in postpartum estrus produced larger, heavier litters than females conceiving after a rest period between litters. In contrast, limiting the energy intake by direct food restriction led to reduced pup mass at birth (Laurien‐Kehnen & Trillmich, 2004).Whatever the cause of the higher postpartum fecundity, this finding clearly speaks against a short‐term physiological cost of reproductive effort.

This argues against the interpretation that the allocation to current reproduction constrains the energy available for somatic maintenance and future reproduction as also found by Descamps, Boutin, McAdam, Berteaux, and Gaillard (2009) for North American red squirrels (*T. hudsonicus*). In their long‐term study on free‐living squirrels, they found no effect of naturally massively varying food abundance (seeds in cones of white spruce) on reproduction and could not detect any trade‐off between current and future reproduction, but found survival costs for young and senescent females. In our case, the low effect of allocation away from reproduction may be caused by the ad lib condition under which the animals lived. When food resources are essentially unlimited, animals may be able to supply energy and nutrients to all essential functions simultaneously without being forced into trade‐offs. If so, this would suggest that energetic adjustments to seasonality are more firmly programmed than the allocation trade‐offs involved in reproduction.

Our experiment demonstrates that the precocial cavies like larger mammals appear to protect maternal condition and survival while reproducing, yet live fast in terms of entering reproduction early and, once mature, reproducing potentially without interruption. In general, we found only minor costs of continuous reproduction. This may be achieved by shifting potential costs of reproduction late in life to the offspring by increasing their mortality and decreasing their growth rate. Similar to the findings on North American red squirrels, our study demonstrates that the expression of costs of reproduction may not primarily be a consequence of body size or extrinsic mortality but may also depend to a major extent on internal physiological regulation of allocation decisions in line with the life history of the species.

## CONFLICT OF INTEREST

The authors have no conflict of interest to declare.

## AUTHOR CONTRIBUTIONS

FT conceived the study, wrote a first draft of the manuscript, and contributed to collection of the data. EG collected most of the data. AG contributed greatly to writing and did the majority of the statistical analyses.

## Data Availability

The data used for the preparation of this manuscript are deposited in Dryad https://doi.org/10.5061/dryad.5k1b239.
