# Supplementary material for: Senescence and costs of reproduction in the life history of a small precocial species
Source: Ecol Evol. 2019 May 29;9(12):7069–79. doi: 10.1002/ece3.5272 (PMC6662319; doi:10.1002/ece3.5272)
Supplement: Supplementary file 1 [file ECE3-9-7069-s001.docx]

**Supplementary Material**

**Table S1:** Individual reproductive characteristics of females in the continuously reproducing (CR) and the intermittently reproducing (IR) group. Age at death (days) >1095 means that the female survived to the end of the experiment. Mean reproductive effort and mean litter size are calculated including ascribed pups from simultaneous parturitions of 2 females within one enclosure.

| **maternal ID** | **maximum mass in g** | **mean reproductive effort** | **number of litters** | **mean litter size** | **age at death (days)** | **experimental condition** |
| --- | --- | --- | --- | --- | --- | --- |
| E0EE | 608 | 0.30 | 16 | 2.44 | >1095 | CR |
| AC74 | 584 | 0.37 | 15 | 3.40 | >1095 | CR |
| DB79 | 658 | 0.33 | 15 | 3.07 | >1095 | CR |
| BDE6 | 531 | 0.35 | 8 | 2.75 | 627 | CR |
| F9B8 | 598 | 0.30 | 16 | 2.50 | >1095 | CR |
| E0FE | 644 | 0.27 | 6 | 2.33 | 495 | CR |
| CE93 | 728 | 0.35 | 17 | 3.29 | >1095 | CR |
| E7D4 | 547 | 0.28 | 4 | 2.00 | 299 | CR |
| 8FC4 | 752 | 0.27 | 16 | 2.63 | >1095 | CR |
| 2658 | 929 | 0.34 | 16 | 4.13 | >1095 | CR |
| 1271 | 679 | 0.39 | 16 | 3.63 | >1095 | CR |
| 92CF | 758 | 0.36 | 9 | 3.56 | 675 | CR |
| B2EC | 709 | 0.33 | 11 | 3.09 | 792 | CR |
| 5349 | 888 | 0.38 | 15 | 4.00 | >1095 | CR |
| 32B2 | 733 | 0.32 | 14 | 3.00 | 923 | CR |
| BE47 | 725 | 0.35 | 16 | 3.44 | >1095 | CR |
| 18B4 | 636 | 0.27 | 11 | 2.55 | >1095 | CR |
|  | | | | | | |
| E763 | 424 | 0.30 | 5 | 2.60 | 565 | IR |
| 7B1D | 634 | 0.32 | 12 | 3.08 | 949 | IR |
| E60C | 702 | 0.35 | 13 | 3.38 | >1095 | IR |
| E83E | 658 | 0.36 | 8 | 4.00 | 628 | IR |
| 0131 | 663 | 0.29 | 13 | 2.54 | >1095 | IR |
| C626 | 773 | 0.31 | 12 | 3.23 | 1056 | IR |
| E07B | 611 | 0.37 | 13 | 3.69 | >1095 | IR |
| AF5A | 628 | 0.32 | 13 | 3.00 | >1095 | IR |
| AF0E | 609 | 0.30 | 12 | 2.33 | >1095 | IR |
| 91A7 | 673 | 0.37 | 11 | 3.36 | 875 | IR |
| 9681 | 619 | 0.39 | 5 | 3.00 | 427 | IR |
| BC67 | 754 | 0.41 | 11 | 4.45 | >1095 | IR |
| A135 | 629 | 0.29 | 14 | 3.00 | >1095 | IR |
| 53CF | 660 | 0.33 | 12 | 2.75 | >1095 | IR |
| 1B5C | 739 | 0.23 | 7 | 2.43 | >1095 | IR |
| 70DE | 595 | 0.36 | 11 | 3.00 | >1095 | IR |
| A30C | 732 | 0.28 | 12 | 2.75 | >1095 | IR |
| 94C7 | 692 | 0.35 | 13 | 3.08 | >1095 | IR |

**Figure S1:** Daily maximal, mean and minimal temperatures in the outdoor enclosures in 2006. Temperature was measured every 30 min.
