# Supplementary material for: Senescence and costs of reproduction in the life history of a small precocial species
Source: Ecol Evol. 2019 May 29;9(12):7069–79. doi: 10.1002/ece3.5272 (PMC6662319; doi:10.1002/ece3.5272)
Supplement: Supplementary file 2 [file ECE3-9-7069-s002.zip › Figure 2 caption.docx]

**Figure S2:** Effects of maternal age on body mass of continuously (CR) and intermittently (IR) reproducing females. Crosses and dotted lines refer to IR females, black dots and solid lines to CR females. Grey bands around the lines represent 95% - confidence intervals of regression lines.
