# Supplementary figures and images for: Senescence and costs of reproduction in the life history of a small precocial species
Source: Ecol Evol. 2019 May 29;9(12):7069–79. doi: 10.1002/ece3.5272 (PMC6662319; doi:10.1002/ece3.5272)

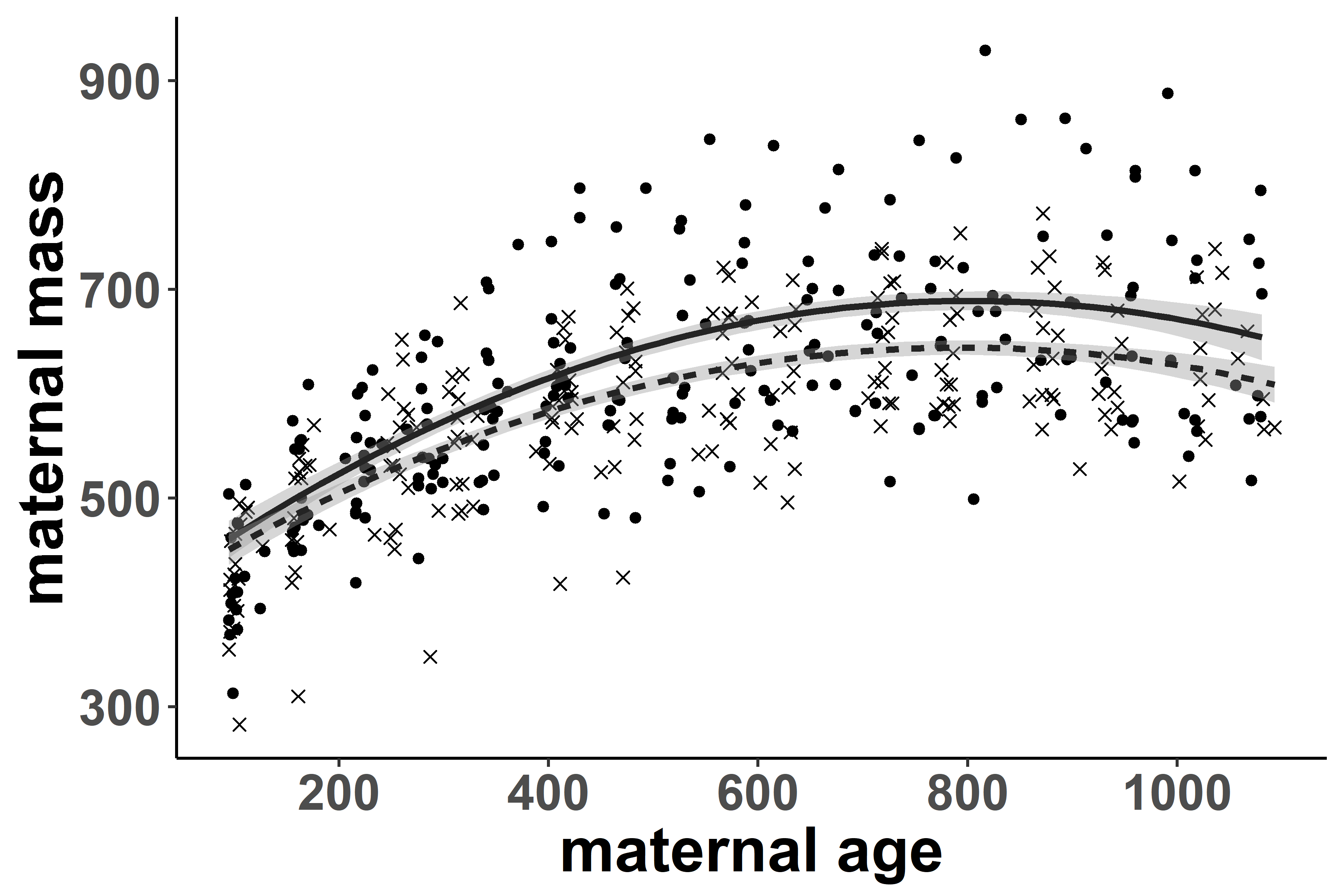

Supplement: Supplementary file 2 [file ECE3-9-7069-s002.zip › Figure_S2_comp.tiff]

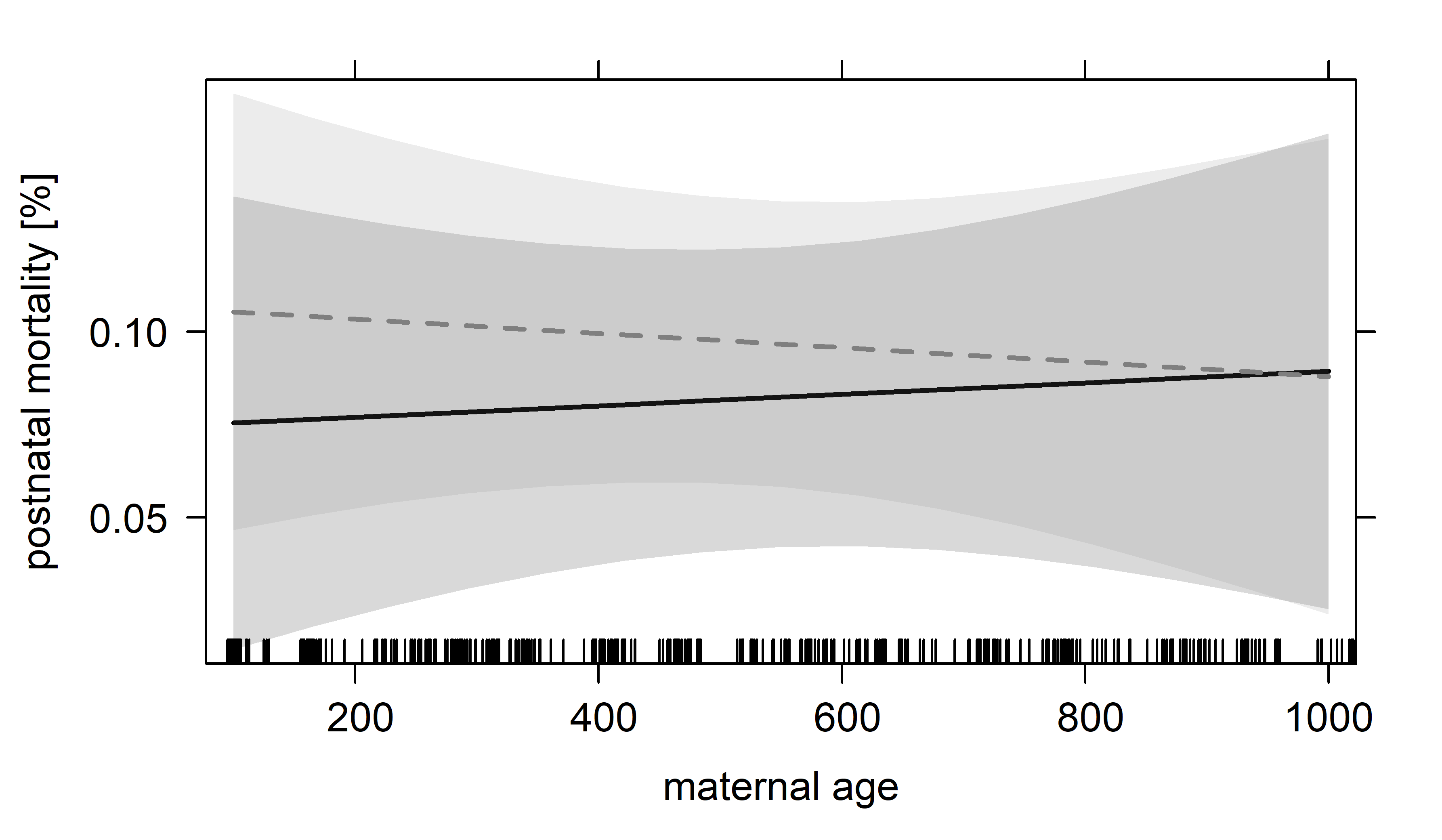

Supplement: Supplementary file 3 [file ECE3-9-7069-s003.zip › Figure_S3_B_comp.tiff]

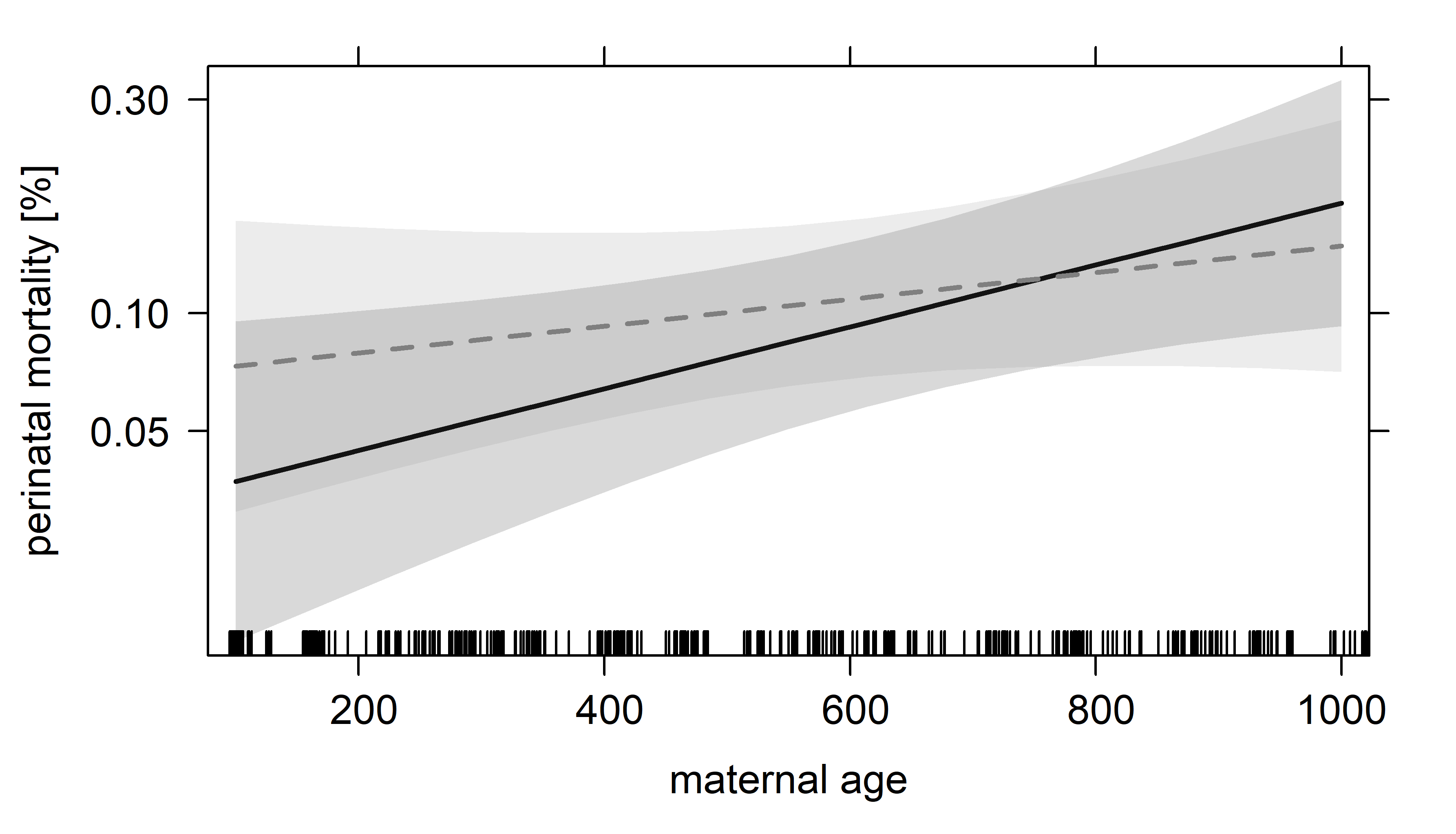

Supplement: Supplementary file 3 [file ECE3-9-7069-s003.zip › Figure_S3_A_comp.tiff]
