# Supplementary material for: Senescence and costs of reproduction in the life history of a small precocial species
Source: Ecol Evol. 2019 May 29;9(12):7069–79. doi: 10.1002/ece3.5272 (PMC6662319; doi:10.1002/ece3.5272)
Supplement: Supplementary file 3 [file ECE3-9-7069-s003.zip › Figure 3 caption.docx]

Figure S3. Effects of maternal age on pup perinatal (upper panel) and postnatal (lower panel) mortality did not differ between CR- and IR-groups. Solid line: CR-group. Dashed line: IR-group. Grey bands (dark gray CR; light grey IR) around the lines represent 95% - confidence intervals of predicted estimates.
